# Simultaneous Determination of a Novel PD-L1 Inhibitor, IMMH-010, and Its Active Metabolite, YPD-29B, in Rat Biological Matrices by Polarity-Switching Liquid Chromatography-Tandem Mass Spectrometry: Application to ADME Studies

**DOI:** 10.3389/fphar.2021.677120

**Published:** 2021-06-21

**Authors:** Jianwei Jiang, Xiaowen Zou, Yuke Liu, Xiao Liu, Kai Dong, Xiaoqing Yao, Zhiqiang Feng, Xiaoguang Chen, Li Sheng, Yan Li

**Affiliations:** ^1^ Department of Drug Metabolism, Institute of Materia Medica, Chinese Academy of Medical Sciences and Peking Union Medical College, Beijing, China; ^2^ Beijing Key Laboratory of Non-Clinical Drug Metabolism and PK/PD Study, Institute of Materia Medica, Chinese Academy of Medical Sciences and Peking Union Medical College, Beijing, China; ^3^ Beijing Key Laboratory of Active Substances Discovery and Drug Ability Evaluation, Institute of Materia Medica, Chinese Academy of Medical Sciences and Peking Union Medical College, Beijing, China; ^4^ State Key Laboratory of Bioactive Substance and Function of Natural Medicines, Institute of Materia Medica, Chinese Academy of Medical Sciences and Peking Union Medical College, Beijing, China; ^5^ Tianjin Chase Sun Pharmaceutical Co. LTD, Tianjin, China

**Keywords:** PD-L1 inhibitor, IMMH-010, YPD-29B, rat biological matrices, LC-MS/MS, ADME studies.

## Abstract

IMMH-010 is a prodrug of YPD-29B, which is a novel PD-L1 inhibitor. A specific and sensitive LC-MS/MS method with polarity switching was developed and validated for the simultaneous determination of IMMH-010 and YPD-29B in rat plasma, liver, brain, urine and fecal samples. Method validation was investigated to demonstrate the lower limit of quantification linearity, precision and accuracy, matrix effect and recovery, stability and dilution reliability for IMMH-010 and YPD-29B. This validated method was successfully applied to investigate the pharmacokinetics, tissue distribution, and excretion of IMMH-010 and YPD-29B in rats. After oral administration of IMMH-010 maleate to rats, IMMH-010 was rapidly and extensively converted to the active metabolite YPD-29B. The areas under the plasma concentration-time curve (AUC) of IMMH-010 and YPD-29B were proportional to the dose in the range of 10–100 mg/kg. IMMH-010 was primarily distributed in the adrenal gland, lymph nodes, heart, liver and spleen. YPD-29B was mainly observed in the liver, lymph, kidney, and lung. Approximately 28.81% of the IMMH-010 dose was recovered in the urine and feces within 72 h, including unchanged IMMH-010 (7.99%) and YPD-29B (20.82%). The results of this study may be useful as a reference for further development of IMMH-010 and PD-L1 inhibitors.

**Clinical Trial Registration**: [https://clinicaltrials.gov/ct2/show/NCT04343859?term=IMMH-010&draw=2&rank=1], identifier [NCT04343859]."

## Introduction

Cancer immunotherapy, which boosts the body’s immune system to induce an antitumor response, is a revolutionary anticancer strategy that has become a main focus of oncology research (Yang, 2015; Hegde et al., 2020). One of the key targets of cancer immunotherapy is the programmed cell death protein 1 (PD-1)/programmed cell death one ligand 1 (PD-L1) checkpoint pathway. Blocking the PD-1/PD-L1 pathway can promote the antitumor immune response and inhibit tumor growth (Xu-Monette et al., 2017; Gong et al. et al., 2018; Lin et al., 2020; Cheung et al., 2019). Since 2014, ten PD-1/PD-L1 inhibitors have been approved for marketing and have been shown to be very effective in treating certain types of tumors. However, all of the PD-1 and PD-L1 inhibitors on the market are monoclonal antibody drugs. Because antibody drugs have disadvantages, including the need for intravenous injection, poor stability, immunogenicity and high cost, developing PD-1/PD-L1 inhibitors is worthwhile (Liu et al., 2020).

IMMH-010, 2-[4-(2-bromo-biphenyl-3-ylmethoxy)-5-chloro-2-(pyridin-3-ylmethoxy)- benzylamino]-3-hydroxy-propionic acid isopropyl ester, can be hydrolyzed at the ester bond to produce the active metabolite YPD-29B. A pharmacological study demonstrated that YPD-29B could effectively block the binding of PD-1 and PD-L1 in a homogeneous time-resolved fluorescence (HTRF) protein-protein interaction assay, and the IC_50_ < 10^−13^ M (patent: CN109153670). IMMH-010 showed significant antitumor activity in various carcinoma xenograft models and entered phase I clinical trials with trial number NCT04343859 (https://clinicaltrials.gov/ct2/show/NCT04343859?term=IMMH-010&draw=2&rank=1). Investigation of pharmacokinetic (PK) is an essential part of drug development, and a reliable analysis method is the basis of PK research. The objective of the present study was to explore a rapid, simple, sensitive and accurate LC-MS/MS method for the simultaneous determination of IMMH-010 and its active metabolite YPD-29B in rat biological matrices. This method was then successfully applied to study the ADME of IMMH-010 in rats and can provide useful information for clinical trials.

## Materials and Methods

### Chemicals

IMMH-010, IMMH-010 maleate, YPD-29B, IMM-H008BP (internal standard, IS_1_), and IMM-H008B (IS_2_) (all compounds with purity>99%) were provided by the Department of Chemosynthesis, Institute of Materia Medica, Chinese Academy of Medical Sciences and Peking Union Medical College. HPLC-grade methanol was obtained from Merck (Darmstadt, Germany). Ammonium acetate was purchased from Dikma (California, United States). Heparin sodium and sodium fluoride (NaF) were purchased from Sigma-Aldrich (Missouri, United States). Water was purified by a Milli-Q ultrapure water system (Merck, Darmstadt, Germany).

### Instrumentation and Analytical Conditions

The LC-MS/MS system consisted of an LC-30A unit (Shimadzu, Japan) and an API 4000 mass spectrometer (AB SCIEX, United States). Chromatographic separation of the 8 μL samples was conducted on a Zorbax SB-C18 column (3.5 μm, 2.1 mm × 100 mm, Agilent, United States) in an oven at 35°C. The mobile phases were water with 1 mM ammonium acetate (phase A) and methanol (phase B) pumped at 0.3 ml/min. The elution conditions were as follows: 70% B, held for 2.8 min, increased to 100% B within 0.3 min, held for 3.7 min, returned to 70% B within 0.1 min and equilibrated for 5.1 min. The total chromatographic separation time was 12 min.

Detection was performed on a triple quadrupole mass spectrometer in multiple reaction monitoring (MRM) mode with electrospray ionization (ESI) under the following conditions: positive ion mode for IMMH-010 and IS_1_ and negative ion mode for YPD-29B and IS_2_. The MS parameters in positive mode were as follows: The ion spray voltage was maintained at 4500 V, the temperature at 550°C, the CAD gas at 10 psi, the CUR gas at 25 psi, gas1/gas2 at 50 psi, the declustering potential (DP) at 70 V for IMMH-010 and IS_1_, and the collision energy (CE) at 20 V for IMMH-010 and 23 V for IS_1_. The mass transition ion pairs were 641.3 → 494.2 for IMMH-010 and 664.4 → 517.5 for IS_1_. The MS parameters in negative mode were as follows: The ion spray voltage was maintained at -4500 V, the temperature at 550°C, the CAD gas at 12 psi, the CUR gas at 10 psi, gas 1/gas 2 at 80/60 psi, the DP at −110 V for YPD-29B and −103 V for IS_1_, and the CE at −45 V for YPD-29B and −53 V for IS_2_. The mass transition ion pairs were 597.1 → 154.8 for YPD-29B and 620.0 → 154.7 for IS_2_.

### Preparation of Stock and Working Solutions

Stock solutions of IMMH-010 (2.30 and 3.20 mg/ml), YPD-29 B (2.28 and 2.84 mg/ml), IMM-H008BP (IS_1_, 3.20 mg/ml) and IMMH-008B (IS_2_, 3.98 mg/ml) were prepared in DMSO. Gradient dilutions of the stock solutions of IMMH-010 and YPD-29B in acetonitrile were prepared for standard calibrators and quality control (QC). The IS stock solutions were diluted with acetonitrile and then mixed to prepare a working solution containing 1,580 ng/ml IS_1_ and 398 ng/ml IS_2_.

### Preparation of Standards and QC Samples

**Plasma**: IMMH-010 is an ester-containing compound. Our preliminary results demonstrated that IMMH-010 was stable at 4°C in rat plasma containing 50 mM NaF (a broad-spectrum and potent esterase inhibitor). Therefore, heparin sodium and NaF were dissolved with 0.9% w/v sodium chloride to prepare a mixture of 0.5% heparin sodium and 500 mM NaF. All blood was withdrawn into EP tubes containing this mixture (1:9 v/v mixture:blood) followed by plasma separation. The plasma standards for IMMH-010 were prepared at 1, 2, 5, 10, 20, 80, 120 and 200 ng/ml, and those for YPD-29B were prepared at 2.5, 5, 10, 20, 50, 400, 600 and 1,000 ng/ml. QC samples were prepared at 1, 2, 40 and 160 ng/ml for IMMH-010 and 2.5, 5, 200 and 800 ng/ml for YPD-29B.

**Tissue homogenates, urine and feces**: After rats were housed in separated metabolic cages to collect blank urine and feces, blank tissues (liver and brain) were collected, washed with normal saline, blotted dry with filter paper and added to normal saline (w:v = 1:3) for homogenization by a homogenizer. Tissue homogenate standards for IMMH-010 were prepared at 1, 2, 5, 10, 20, 80, 120, and 200 ng/ml, and those for YPD-29B were prepared at 2.5, 5, 10, 20, 50, 400, 600, and 1,000 ng/ml. QC samples were prepared at 1, 2, 40 and 160 ng/ml for IMMH-010 and 2.5, 5, 200, and 800 ng/ml for YPD-29B. The urine standards for IMMH-010 were prepared at 0.5, 1, 2.5, 10, 20, 40, and 60 ng/ml, and those for YPD-29B were prepared at 1, 3, 5, 20, 40, 50, and 80 ng/ml. QC samples were prepared at 0.5, 1, 5, and 50 ng/ml for IMMH-010 and 1, 3, 10, and 60 ng/ml for YPD-29B. Fecal standards for IMMH-010 were prepared at 1, 2, 5, 10, 20, 50, 60, and 100 ng/ml, and those for YPD-29B were prepared at 2.5, 5, 10, 20, 50, 400, 600, and 1,000 ng/ml. QC samples were prepared at 1, 2, 40, and 80 ng/ml for IMMH-010 and 2.5, 5, 200, and 800 ng/ml for YPD-29B.

### Sample Preparation

All samples before precipitation were maintained at 4°C. All blood was withdrawn into EP tubes containing a mixture of 0.5% heparin sodium and 500 mM NaF (1:9 v/v mixture:blood) and then centrifuged at 5,000 rpm for 10 min to collect plasma. The tissues were washed with normal saline, blotted dry with filter paper and homogenized with normal saline at 1:3 (w/v). Urine samples were directly diluted 10-fold in methanol. The air-dried fecal samples were homogenized with DMSO at 1:6 (w/v), followed by 100-fold dilution with methanol. For all the prepared samples, a 30-μL aliquot was added to 60 μL of a mixed IS solution. Then, the mixture was vortexed and centrifuged at 14,000 rpm for 5 min, and 8 μL of the supernatant was analyzed by LC-MS/MS.

### Method Validation

#### Selectivity

A selectivity assay was performed to identify whether there were any interferences in the six blank rat matrix samples (plasma, tissues, urine and feces) from separate sources that could influence the analysis of IMMH-010, YPD-29B and the IS. The peak area of interferences eluting at the retention times (RTs) of analytes and the IS was required to be less than 20% of the peak area in the lower limit of quantification (LLOQ) samples and less than 5% of the IS peak area.

#### Linearity

Linearity was evaluated from calibration curves with seven or eight concentrations for IMMH-010 and YPD-29 B. The linearity of a calibration curve was obtained by plotting the peak area ratio (analyte/IS) vs. the nominal concentration using a linearly weighted 1/*x* ^2^ (*x* = concentration) least squares regression.

#### Precision and Accuracy

The relative standard deviation (RSD, %) and the relative error (RE, %) were used to evaluate precision and accuracy, respectively. The intra- and inter-day precision and accuracy of the matrix were calculated with LLOQ samples and QC samples at low, intermediate and high levels each day (*n* = 6) on three separate days. The intra- and inter-day precision and accuracy were within ±15% for all QC samples and within ±20% for all LLOQ samples, indicating the established method have good reproducibility and accuracy.

#### Matrix Effect and Recovery

The matrix effect in plasma samples was evaluated as the IS-normalized matrix factor (MF). The MF is the ratio of the peak area of the analytes in a deproteinized blank matrix spiked with analytes to the peak area of the analytes in acetonitrile solution. The IS-normalized MF is the ratio of MF_analyte_ to MF_IS_, where MF_analyte_ and MF_IS_ are the MF of the analyte and internal standard, respectively.

The recovery was investigated by comparing the peak responses in blank matrix samples spiked with analyte to those in post-extracted samples spiked with analyte. Matrix effect and recovery were both evaluated at three concentration levels (high, intermediate and low QC) (*n* = 6).

#### Stability

For short-term and long-term stability, plasma samples were kept at 4°C for 4 h and −20°C for 3 months, respectively. To evaluate freeze-thaw stability, samples were frozen at −20°C for 24 h and thawed at 4°C for three cycles. For stability of analytical procedures, the prepared samples were stored in an autosampler at 10°C for 48 h before analysis. Stability assays of IMMH-010 and YPD-29B were performed at two QC levels (high and low QC) (*n* = 5).

#### Dilution Reliability

Plasma samples with 800 ng/ml IMMH-010 and 4,000 ng/ml YPD-29 B were diluted 1:4 (v/v) with blank plasma, and the precision and accuracy were determined (n = 5).

### Method Application to ADME Studies

#### Animals

Female Sprague-Dawley rats (200 ± 20 g, Vital River, Beijing, China) were kept in standard environmental conditions. Rats were fasted overnight with free access to water and randomly assigned to different groups before the experiments. All animal protocols were approved by the Institute Animal Care and Welfare Committee of the Institute of Materia Medica, Chinese Academy of Medical Sciences and Peking Union Medical College.

#### PK Study

IMMH-010 maleate was suspended in 0.5% sodium carboxymethyl cellulose. The rats were intragastrically (i.g.) administered IMMH-010 maleate at doses of 10, 30, and 100 mg/kg. Blood samples were withdrawn via the orbital plexus into heparinized tubes with NaF at 5, 15, and 30 min and 1, 2, 4, 6, 8, 12, 24, 36, and 48 h. Plasma was immediately prepared by centrifugation at 5,000 rpm for 10 min. All samples were stored at −20°C prior to analysis. The PK parameters of IMMH-010 and YPD-29B were calculated using the Linear Trapezoidal with Linear Interpolation method by WinNonlin software version 6.3 based on a noncompartmental model (Pharsight Corporation, Mountain View, United States ).

#### Tissue Distribution Study

The tissue distribution study was performed 15 min, 30 min, 4 h and 12 h after i. g. administration of 10 mg/kg IMMH-010 maleate. Blood was collected and centrifuged at 5,000 rpm for 10 min. The heart, liver, brain, spleen, lung, kidney, adrenal gland, thymus, and lymph were harvested immediately. The tissues were washed with normal saline and blotted dry with filter paper. All samples were stored at -20°C before analysis.

#### Excretion Study

After i. g. administration of IMMH-010 maleate (10 mg/kg), rats were housed in separate metabolite cages to collect their urine and feces: 0–12, 12–24, 24–36, 36–48, and 48–72 h for urine and 0–24, 24–48, and 48–72 h for feces. Fecal samples were dried in air. Urine samples were stored at −20°C prior to analysis.

### Statistical Analysis

All data are expressed as the mean ± standard deviation (SD). The profiles of the mean blood concentration vs. time were plotted by GraphPad Prism 5 software (San Diego, United States).

## Results and Discussion

### Method Development

**FIGURE 1:**
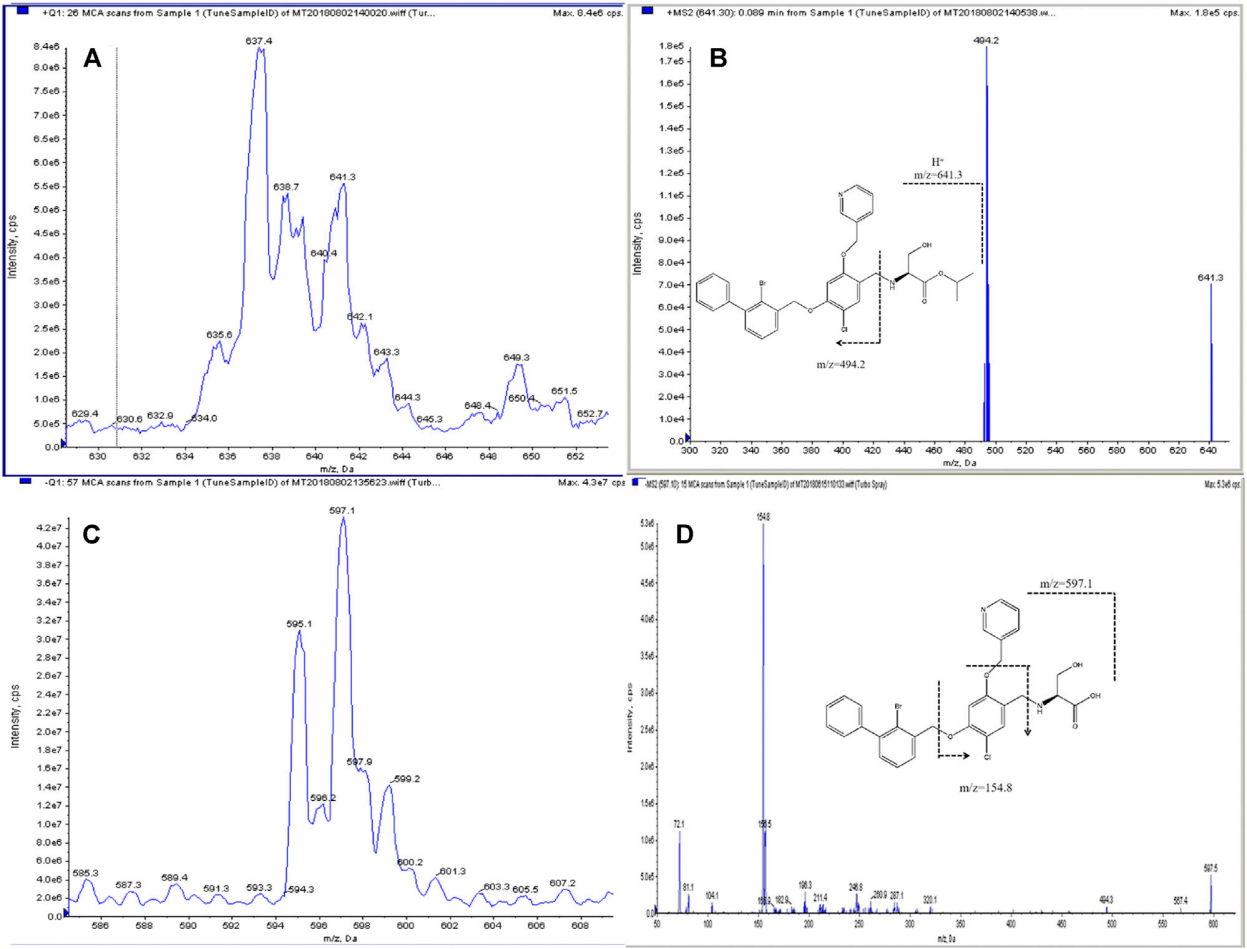
Structure and product ion spectra with proposed MS fragmentations: parent ion **(A)** and daughter ion **(B)** of IMMH-010 and parent ion **(C)** and daughter ion **(D)** of YPD-29B.

Although IMMH-010 and YPD-29 have similar chemical structures, IMMH-010, with a secondary amine group in its chain, exhibited a stronger signal intensity in positive ion mode, while YPD-29B, containing a carboxyl group, had a greater response in negative ion mode. Therefore, the positive-negative polarity switching mode was applied for the simultaneous determination of IMMH-010 and YPD29B. The structural analogs IMM-H008BP (IS_1_) and IMM-H008B (IS_2_) were selected as the ISs of IMMH-010 and YPD-29B, respectively. In full-scan mode, the protonated molecular ion [M + H+2]^+^ was chosen as the parent ion for IMMH-010 and IS_1_, and the deprotonated form [M-H+2]^-^ for YPD-29B and IS_2_. The predominant transitions for quantification were: *m*/*z* 641.3→494.2 for IMMH-010, *m*/*z* 664.4→517.5 for IS_1_, *m*/*z* 597.1→154.8 for YPD-29B and *m*/*z* 620.0→154.7 for IS_2_. Then, the DP, gas pressure and CE were optimized. The structures and product ion spectra of IMMH-010, YPD-29B and IS are presented in Figure 1.

To measure both positive ions and negative ions in one method, it was necessary to divide the chromatogram into two sections based on the RTs of the analytes and apply the positive-negative polarity switching mode. Therefore, the first challenge was to optimize the chromatographic conditions to fully separate the compounds analyzed with different scan modes. We selected the column and optimized the mobile phase composition and elution gradient slope according to the retention behavior of the analytes.

Compared with the acetonitrile-water system, the methanol-water system resulted in a stronger separation capability and better signal response of the analytes, so a methanol gradient was applied. To optimize peak shape and separation, different types of columns were tested, including Zorbax C8, Zorbax C18, CAPCELL PAK ADME and Atlantis T3 columns. Among them, the Zorbax C18 column provided the best resolution and peak shape. Subsequently, the effects of different concentrations of mobile phase additives (ammonia, formic acid and ammonium acetate) on peak shape and analyte ionization were evaluated. Ammonium acetate (1 mM) improved the peak shape of YPD-29B, while the ionization of YPD-29B was suppressed when using 0.1% formic acid. Therefore, the mobile phase consisted of water and methanol with 1 mM ammonium acetate. Furthermore, when the initial concentration of methanol was 60%, the analytes could be separated from each other, and increasing the initial concentration of methanol resulted in increased resolution and decreased sensitivity. After comprehensively considering the resolution and sensitivity, a gradient starting with 70% methanol was employed, and all analytes achieved narrow peak widths and efficient separation, ensuring successful positive/negative polarity-switching analysis. Ultimately, in the first 5 min of the chromatographic run, YPD-29B and IS_2_ were detected in MRM negative ion mode; then, the polarity of the instrument was switched to positive ion mode, and IMMH-010 and IS_1_ were monitored.

Due to the low ionization efficiency of YPD-29B in negative ion mode, efforts were made to optimize not only the HPLC separation conditions but also the sample preparation procedures, and liquid-liquid extraction, solid-phase extraction and various precipitation conditions were evaluated. When using Oasis HLB and Sep-Pak C18 Vac cartridges for solid-phase extraction, the recovery of IMMH-010 was extremely low. When using ether, ethyl acetate and dichloromethane for liquid-liquid extraction, the recovery of YPD-29B was too low to detect the two analytes simultaneously. The signals of IMMH-010 and YPD-29B after protein precipitation were decreased due to the matrix effect, but considering the advantages of protein precipitation (simplicity, minimal sample loss, and inexpensive reagents), the conditions for protein precipitation using 20% trichloroacetic acid, methanol and acetonitrile were further optimized. The maximum sensitivity was achieved only when two volumes of acetonitrile were added. In addition, the response of YPD-29B was proportional to the injection volume below 8 μL, without peak broadening. Therefore, samples were precipitated with two volumes of acetonitrile, and 8 μL of supernatant was injected for quantification.

### Method Validation

#### Selectivity

**FIGURE 2:**
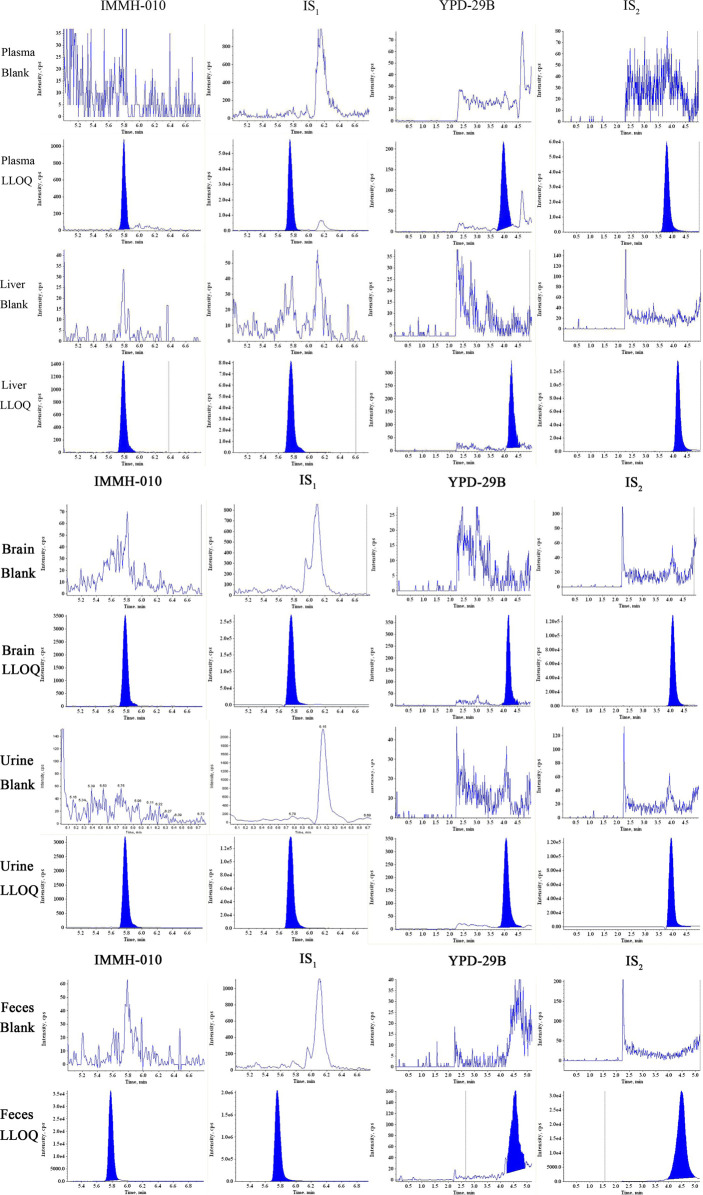
Typical MRM chromatograms of IMMH-010, IS_1_, YPD-29B and IS_2_ in plasma, liver, brain and urine and feces obtained from blank sample and LLOQ samples.

Figure 2 shows the representative LC-MS/MS chromatogram obtained from a blank sample, blank sample spiked with analytes at the LLOQ. The RTs of IMMH-010 and IS_1_ were 5.80 and 5.76 min, respectively, and those of YPD-29B and IS_2_ were 3.95 and 3.80 min, respectively. There were no significant interferences from rat plasma at the RTs of the two analytes or ISs.

#### Linearity and Carryover

As shown in Table 1, IMMH-010 and YPD-29B showed good linearity over ranges of 1–200 ng/ml and 2.5–1,000 ng/ml for plasma and tissue (liver and brain), 1–100 ng/ml and 2.5–1,000 ng/ml for feces, and 0.5–60 ng/ml and 1–80 ng/ml for urine samples, all with determination coefficients (*r* ^2^) greater than 0.99. In the plasma, tissue and feces matrices, the LLOQs of IMMH-010 and YPD-29 B were 1 ng/ml and 2.5 ng/ml, and the ULOQs of IMMH-010 and YPD29B were 200 ng/ml (100 ng/ml for feces only) and 1,000 ng/ml, respectively. In the urine matrix, the LLOQs of IMMH-010 and YPD-29B were 0.5 ng/ml and 1 ng/ml, and the ULOQs of IMMH-010 and YPD29B were 60 ng/ml and 80 ng/ml, respectively. After injection of the ULOQ samples, blank samples were analyzed, and IMMH-010 and YPD-29B could not be found in the blank samples. Therefore, the carryover was negligible.

**TABLE 1:** The regression equations and LLOQs of IMMH-010 and YPD-29B in biological matrices.

Biological matrices		IMMH-010			YPD-29B	
	Range (ng/ml)	Regression equation	LLOQ (ng/ml)	Range (ng/ml)	Regression equation	LLOQ (ng/ml)
Plasma	1.0–200	Y = 0.0071X+0.00129 (r = 0.9978)	1.0	2.5–1,000	Y = 0.0015X-0.00008 (r = 0.9981)	2.5
Brain	1.0–200	Y = 0.0112X+0.00676 (r = 0.9973)	1.0	2.5–1,000	Y = 0.00124X+0.000497 (r = 0.9992)	2.5
Liver	1.0–200	Y = 0.00851X+0.00147 (r = 0.9994)	1.0	2.5–1,000	Y = 0.00165X+0.000486 (r = 0.9993)	2.5
Urine	0.5–60	Y = 0.0122X+0.00354 (r = 0.9980)	0.5	1–80	Y = 0.00144X+0.001 (r = 0.9974)	1.0
Feces	1.0–100	Y = 0.0116X+0.00284 (r = 0.9980)	1.0	2.5–1,000	Y = 0.00162X+0.000587 (r = 0.9984)	2.5

#### Precision and Accuracy

The intra-day and inter-day precision and accuracy values of IMMH-010 and YPD-29B in plasma at the LLOQs and three QC levels are shown in Table 2. The intra-day and inter-day precision (RSD, %) of IMMH-010 and YPD-29B were within 12.5%, and the accuracy (RE, %) was within 12.7%. The intra-day precision and accuracy values of IMMH-010 and YPD-29B in tissues (brain and liver), urine and feces at the LLOQs and three QC levels are shown in Table 3. The intra-day precision (RSD, %) of the analytes was within 12.0%, and the accuracy (RE, %) was within 14.2% for all QC samples and within 17.6% for LLOQ samples.

**TABLE 2:** Intra-day (*n* = 6) and inter-day (*n* = 18) precision and accuracy of IMMH-010 and YPD-29B in rat plasma.

Analyte	Spiked (ng/ml)	Intra-day		Inter-day	
		Precision (RSD, %)	Accuracy (RE, %)	Precision (RSD, %)	Accuracy (RE, %)
IMMH-010	1	12.5	1.6	10.5	−0.5
	2	6.3	12.7	6.6	7.6
	40	5.3	−1.0	4.6	3.1
	160	3.3	−0.1	2.9	−1.2
YPD-29B	2.5	3.0	0.5	6.0	0.7
	5	3.6	−3.6	5.9	−3.3
	200	2.4	1.5	2.9	3.6
	800	1.4	−0.1	3.2	5.0

**TABLE 3:** Intra-day precision and accuracy of IMMH-010 and YPD-29B in liver, brain, urine and feces (*n* = 6).

Matrix	Spiked (ng/ml)	IMMH-010		Spiked (ng/ml)	YPD-29 B	
		Precision (RSD, %)	Accuracy (RE, %)		Precision (RSD, %)	Accuracy (RE, %)
Liver	1	3.0	−3.4	2.5	2.9	2.0
	2	3.8	2.6	5	3.1	−2.3
	40	2.8	5.5	200	1.9	4.5
	160	4.9	−1.6	800	2.0	3.5
Brain	1	2.5	17.2	2.5	3.7	0.8
	2	12.0	4.6	5	2.5	−1.1
	40	8.9	−0.4	200	1.6	−0.7
	160	7.2	−3.9	800	2.0	0.4
Urine	0.5	4.3	−10.6	1	4.4	14.0
	1	5.9	1.0	3	4.0	−7.3
	5	2.5	3.4	10	1.2	−14.2
	50	2.4	0.2	60	1.1	4.5
Feces	1	5.1	−6.3	2.5	9.5	17.6
	2	3.5	1.0	5	5.0	−8.2
	40	2.3	7.5	200	1.0	2.0
	80	1.0	4.0	800	0.9	6.8

#### Matrix Effect and Recovery

The matrix effects and recoveries of IMMH-010 and YPD-29B in plasma samples at three QC levels are presented in Table 4. The IS-normalized MFs of IMMH-010 and YPD-29B were 0.529–0.549 and 0.734–0.791, respectively, the coefficient of variation (CV, %) of IS-normalized MF were within 2.9%, suggesting that rat plasma matrix did not affect the quantitation of IMMH-010 or YPD-29 B. The recoveries were 111–114% and 106–111%, respectively, indicating good recoveries from rat plasma.

**TABLE 4:** Internal standard normalization matrix factor and recovery of IMMH-010 and YPD-29B in rat plasma (*n* = 6).

Analyte	Spiked (ng/ml)	IS-normalized MF	Recovery (%)
IMMH-010	2	0.529	111
	40	0.544	114
	160	0.549	112
YPD-29 B	5	0.791	111
	200	0.734	106
	800	0.788	108

#### Stability Study

The stability results are listed in Table 5. The plasma samples were stable at 4°C for 4 h and −20°C for 3 months. Three freeze-thaw cycles did not affect the sample stability. In addition, after precipitation, samples were stable in an autosampler at 10°C for 48 h. All the stability results indicated that IMMH-010 and YPD-29B were stable during the sample preparation, storage and analysis.

**TABLE 5:** Stability of IMMH-010 and YPD-29B in rat plasma (*n* = 5).

Conditions	IMMH-010			YPD-29B		
	Spiked (ng/ml)	Measured conc. (ng/ml)	Accuracy (RE, %)	Spiked (ng/ml)	Measured conc. (ng/ml)	Accuracy (RE,%)
4°C (4 h)	2	2.05 ± 0.10	2.5	5	4.76 ± 0.12	−4.8
	160	173 ± 20	8.1	800	832 ± 8	4.0
Three freeze–thaw cycles	2	2.11 ± 0.22	5.5	5	4.69 ± 0.21	−6.2
	160	157 ± 4	−1.9	800	831 ± 51	3.9
Stored at −20°C (3 months)	2	2.24 ± 0.10	12.0	5	4.87 ± 0.50	−2.6
	160	166 ± 4	3.8	800	748 ± 3	−6.5
At 15°C in auto-sampler (24 h)	2	1.88 ± 0.21	−6.0	5	4.92 ± 0.25	−1.6
	160	148 ± 4	−7.5	800	845 ± 13	5.6
At 15°C in auto-sampler (48 h)	2	2.04 ± 0.07	2.0	5	4.39 ± 0.19	−12.2
	160	154 ± 4	−3.8	800	791 ± 17	−1.1

#### Dilution Reliability

Plasma samples with 800 ng/ml IMMH-010 and 4,000 ng/ml YPD-29B were diluted with blank plasma to yield 160 ng/ml IMMH-010 and 800 ng/ml YPD-29B. The precision and accuracy for the diluted samples are presented in Table 6. All values of RSD and RE were within 15%, suggesting that the determination of the analytes higher than the ULOQ in plasma samples diluted 5-fold was reliable.

**TABLE 6:** Dilution effect of IMMH-010 and YPD-29B in rat plasma (*n* = 5).

Analyte	Spiked (ng/ml)	Dilution factor	Nominal conc. (ng/ml)	Measured conc. (ng/ml)	Precision (RSD,%)	Accuracy (RE, %)
IMMH-010	800	5	160	791 ± 22	2.8	−1.1
YPD-29B	4,000	5	800	3,954 ± 98	2.5	−1.2

### Method Application to ADME Studies

#### PK Study

**FIGURE 3:**
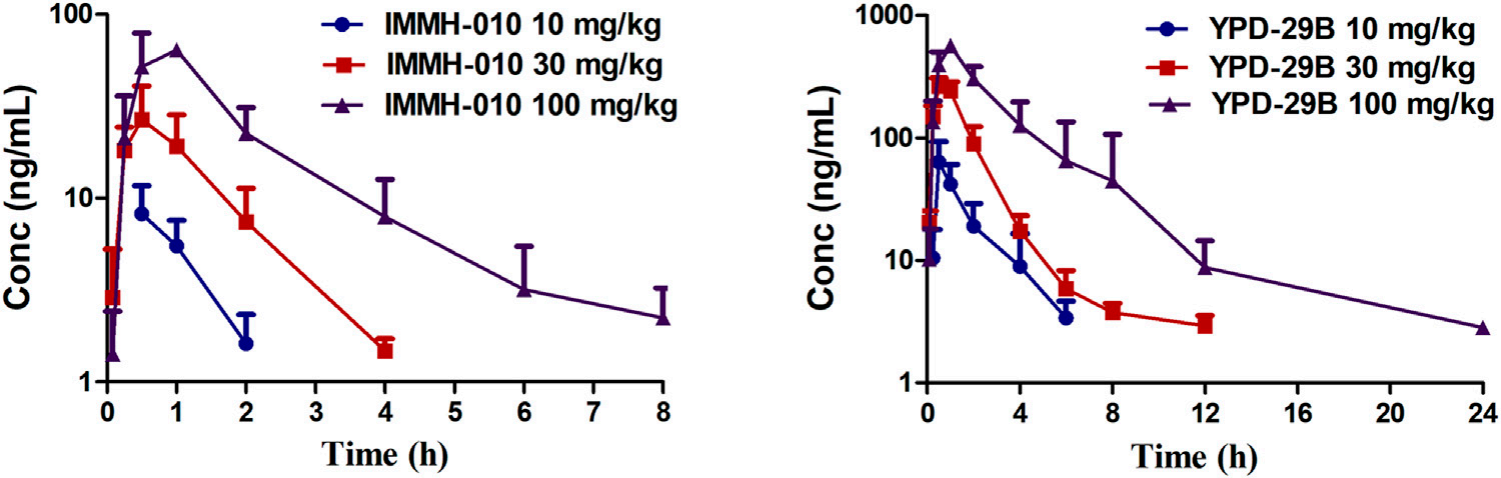
Concentration-time profiles of IMMH-010 and its active metabolites YPD-29B in rats after a single i. g. administration of IMMH-010 maleate at doses of 10, 30, 100 mg/kg (*n* = 5).

**FIGURE 4:**
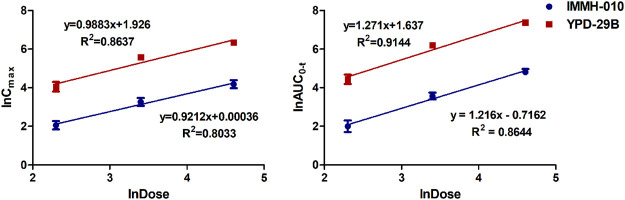
Does proportionality of C_max_ (left) and AUC_0-t_ (right) for IMMH-010 and YPD-29B in plasma with a single i. g. administration of IMMH-010 maleate at doses of 10, 30, 100 mg/kg.

The validated method was successfully applied to PK studies of IMMH-010 and YPD-29B in rats after a single i. g. administration of IMMH-010 maleate at 10, 30 and 100 mg/kg. The blood concentration-time profiles of the two analytes are presented in Figure 3, and the major PK parameters are shown in Table 7. After oral administration, YPD-29B could be detected at 5 min postdose, indicating that YPD-29B was formed rapidly. Both IMMH-010 and YPD-29B reached their peak concentrations (C_max_) at 0.5–1 h. The average plasma elimination half-life (t_1/2β_) of YPD-29B was 1.57–3.65 h. IMMH-010 was extensively converted to YPD-29B, with average AUC ratios (AUC_YPD-29B_/AUC_IMMH-010_) of 11.1, 13.4, and 12.5 for oral doses of 10, 30 and 100 mg/kg, respectively. From the C_max_-dose and AUC_0-t_-dose scatter diagrams in Figure 4, the C_max_ of IMMH-010 and YPD-29B increased proportionally when the dose was increased (*β*_Cmax_ = 0.92, *β*_AUC0-t_ = 1.22 for IMMH-010; *β*_Cmax_ = 0.99, *β*_AUC0-t_ = 1.27 for YPD-29B). The mean residence time (MRT) of IMMH-010 and YPD-29B increased with the dose of IMMH-010, indicating that the pharmacokinetic properties of IMMH-010 exhibited saturation of eliminate in rat.

**TABLE 7:** Pharmacokinetic parameters of IMMH-010 and YPD-29B in rats plasma after a single i.g. administration of IMMH-010 maleate at 10, 30 and 100 mg/kg. Data are expressed as mean ± SD or median (range) (*n* = 5).

Parameters	Units	IMMH-010			YPD-29 B		
		10 mg/kg	30 mg/kg	100 mg/kg	10 mg/kg	30 mg/kg	100 mg/kg
t_1/2β_	h	—	0.98 ± 0.28	1.63 ± 0.49	1.57 ± 1.08	3.65 ± 2.77	3.10 ± 1.76
T_max_	h	0.5 (0.5, 1.0)	0.5 (0.5, 1.0)	0.5 (0.5, 1.0)	0.5 (0.5, 0.5)	0.50 (0.5, 1.0)	1.0 (1.0, 1.0)
C_max_	ng/mL	8.45 ± 3.27	28.4 ± 13.0	71.4 ± 34.0	63.7 ± 29.1	268 ± 43	569 ± 72
AUC_(0-t)_	ng/mL *h	8.50 ± 3.67	37.5 ± 13.5	129 ± 45.6	94.3 ± 46.3	503 ± 110	1,618 ± 336
AUC_(0-∞)_	ng/mL *h	—	44.1 ± 20.6	134 ± 45.7	110 ± 46.6	521 ± 114	1,641 ± 334
MRT_(0-t)_	h	0.80 ± 0.13	1.09 ± 0.21	1.82 ± 0.53	1.5 ± 0.40	1.55 ± 0.14	2.88 ± 0.89
MRT_(0-∞)_	h	—	1.49 ± 0.3	2.11 ± 0.69	2.27 ± 0.96	2.06 ± 0.55	3.12 ± 0.87

AUC area under the concentration-time curve, C_max_ maximum plasma concentration, MRT mean residence time, t_1/2β_ terminal elimination half-life, T_max_ the time taken to reach the maximum concentration.

“-“: those parameters were not calculated because there were only two time-points in elimination phase.

#### Tissue Distribution Study

**FIGURE 5:**
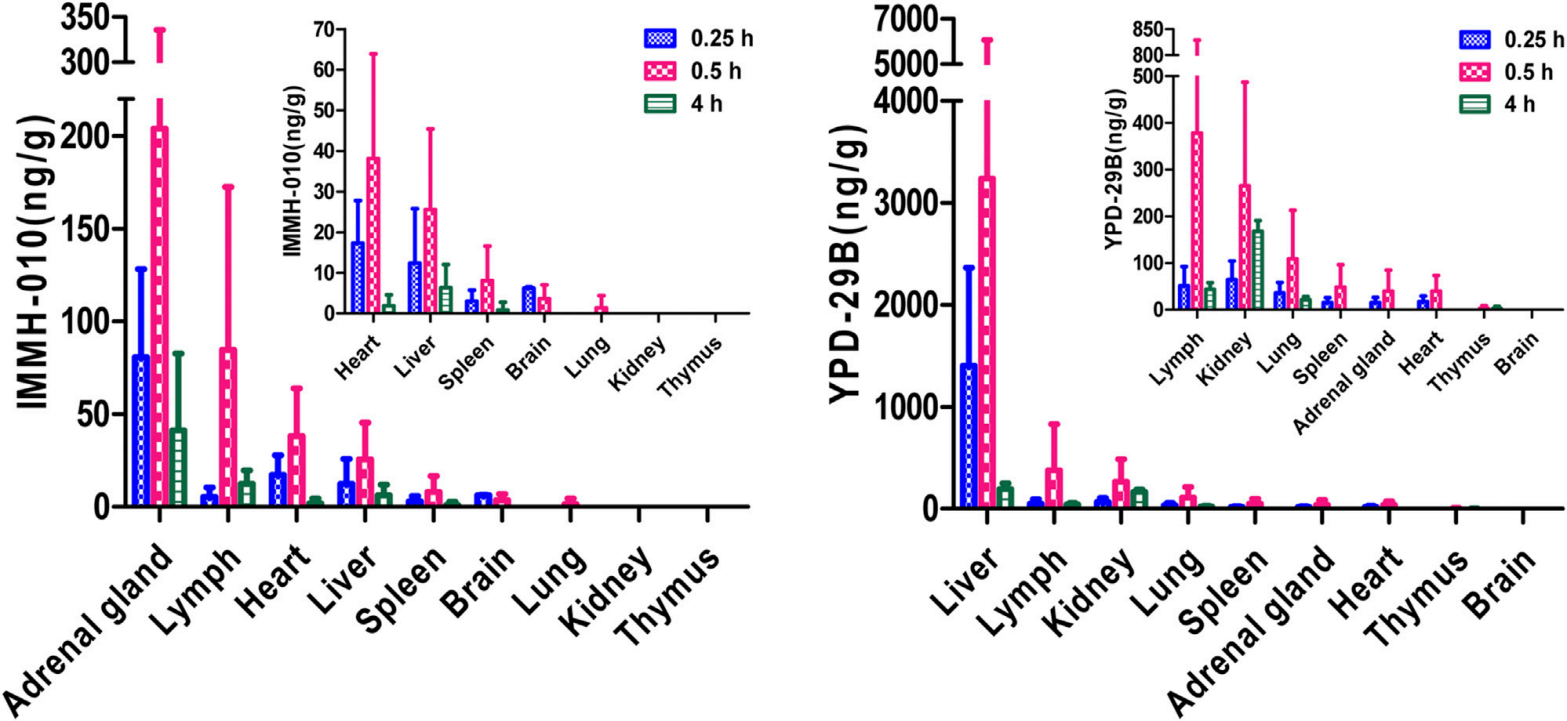
Tissue distribution of IMMH-010 and YPD-29 B in rats after a single i. g. administration of IMMH-010 maleate at 10 mg/kg (*n* = 5).

After oral administration of 10 mg/kg IMMH-010 maleate to rats, the concentrations of IMMH-010 and YPD-29B in plasma and nine different tissues (heart, liver, brain, kidney, spleen, lung, adrenal gland, thymus, and lymph) and their tissue-to-plasma partition (T/P) values are shown in Table 8. As shown in Figure 5, when administered orally, IMMH-010 and YPD-29B were distributed to tissues rapidly and reached their C_max_ values at 30 min postdose. IMMH-010 was distributed mainly in the adrenal gland, followed by the lymph, heart, liver and spleen. For YPD-29B, the highest T/P value was observed in the liver, followed by the lymph, kidney, and lung. The T/P values of the above tissues were greater than unity. The exposure of YPD-29B in the examined tissues was much higher than that of IMMH-010, with the exception of the heart, adrenal gland and brain. At 12 h, the concentrations of IMMH-010 and YPD-29B were below the LLOQ, indicating that the two analytes were quickly eliminated from tissues.

**TABLE 8:** Tissue distribution of IMMH-010 and YPD-29B after i.g. administration of IMMH-010 maleate at 10 mg/kg (*n* = 5).

Tissue	IMMH-010 (ng/ml or ng/g)					YPD-29B (ng/ml or ng/g)					
Time	0.25	T/P	0.5	T/P	4	0.25	T/P	0.5	T/P	4	T/P
Plasma	3.23 ± 2.28	—	3.99 ± 3.45	—	BLQ	32.0 ± 25.2	—	55.7 ± 51.6	—	4.86 ± 1.39	—
Heart	17.3 ± 10.5	5.4	38.1 ± 25.8	9.5	1.92 ± 2.66	17.3 ± 11.9	0.5	39.6 ± 33.7	0.7	BLQ	—
Liver	12.4 ± 13.4	3.8	25.6 ± 19.8	6.4	6.30 ± 5.77	1,403 ± 962	43.8	3,239 ± 2,833	58.2	195 ± 56	40.1
Brain	6.22 ± 0.34	1.9	3.66 ± 3.35	0.9	BLQ	BLQ	—	BLQ	—	BLQ	—
Spleen	2.98 ± 2.77	0.9	8.08 ± 8.51	2.0	0.848 ± 1.896	15.1 ± 10.8	0.5	48.4 ± 47.9	0.9	BLQ	—
Lung	BLQ	—	1.37 ± 3.06	0.3	BLQ	35.7 ± 22.8	1.1	109 ± 104	2.0	21.4 ± 6.8	4.4
Kidney	BLQ	—	BLQ	—	BLQ	64.2 ± 40.4	2.0	265 ± 222	4.8	168 ± 23	34.6
Adrenal gland	80.8 ± 47.4	25.0	204 ± 132	51.1	41.3 ± 6.9	15.3 ± 10.8	0.5	39.7 ± 45.3	0.7	BLQ	—
Thymus	BLQ	—	BLQ	—	BLQ	BLQ	—	2.74 ± 6.12	0.05	2.23 ± 4.99	0.5
Lymph	5.46 ± 5.1	1.7	84.6 ± 87.9	21.2	12.5 ± 7.1	51.5 ± 41.0	1.6	378 ± 451	6.8	44.0 ± 13.9	9.1

BLQ below the lower limit of quantification.

“-“: those parameters were not calculated.

#### Excretion Study

After oral administration of 10 mg/kg IMMH-010 maleate, the excretion of IMMH-010 in feces and urine is shown in Table 9. IMMH-010 was detected in only feces. Approximately 27.64% of the IMMH-010 dose was recovered in the feces within 72 h, including unchanged IMMH-010 (7.99%) and the active metabolite YPD-29B (19.65%). The urinary excretion of metabolite YPD-29B played a minor role, accounting for 1.17% of the dose. Therefore, other metabolites need to be identified.

**TABLE 9:** The excretion of IMMH-010 and YPD-29B from urine and feces after i.g. administration of IMMH-010 maleate (10 mg/kg) (*n* = 5).

	Time (h)	% Of administered dose	
		IMMH-010	YPD-29B
Urine	12	—	0.52 ± 0.15
	24	—	0.63 ± 0.15
	36	—	0.71 ± 0.17
	48	—	0.85 ± 0.36
	72	—	1.17 ± 0.91
Feces	24	6.97 ± 5.69	14.56 ± 8.45
	48	7.99 ± 5.66	19.42 ± 6.85
	72	7.99 ± 5.66	19.65 ± 6.76

“-“: IMMH-010 was not detected in urine.

## Conclusion

A novel and simple polarity-switching LC-MS/MS method was developed and validated to simultaneously determine IMMH-010 and its active metabolite YPD-29B in rat plasma, liver, brain, urine, and fecal samples. This assay was successfully applied to investigate the pharmacokinetics, tissue distribution, and excretion of IMMH-010 and YPD-29B in rats. This is the first paper on the pharmacokinetics of a novel PD-L1 inhibitor. The results of this study may be useful as a reference for further development of IMMH-010 and PD-L1 inhibitors.

## Data Availability

The original contributions presented in the study are included in the article/Supplementary Material, further inquiries can be directed to the corresponding author.
